# Infrared Radiation in the Management of Musculoskeletal Conditions and Chronic Pain: A Systematic Review

**DOI:** 10.3390/ejihpe12030024

**Published:** 2022-03-14

**Authors:** Christos Tsagkaris, Andreas S. Papazoglou, Anna Eleftheriades, Stavros Tsakopoulos, Athanasios Alexiou, Mihnea-Alexandru Găman, Dimitrios V. Moysidis

**Affiliations:** 1 Novel Global Community Educational Foundation, Blacktown 2770, Australia; med3618@edu.med.uoc.gr (C.T.); alexiou@ngcef.net (A.A.); 2 Naval Hospital of Athens, 15561 Athens, Greece; aspapazo@auth.gr; 3 Faculty of Medicine, National and Kapodistrian University of Athens, 11527 Athens, Greece; melefth@uoa.gr; 4 Faculty of Medicine, University of Crete, 71003 Heraklion, Greece; med3699@edu.med.uoc.gr; 5 Faculty of Medicine, “Carol Davila” University of Medicine and Pharmacy, 050474 Bucharest, Romania; 6 Department of Hematology, Center of Hematology and Bone Marrow Transplantation, Fundeni Clinical Institute, 022328 Bucharest, Romania; 7 Faculty of Medicine, Aristotle University of Thessaloniki, 54124 Thessaloniki, Greece

**Keywords:** infrared phototherapy, photodynamic therapy, musculoskeletal conditions, low back pain, osteoarthritis, fibromyalgia, physical therapy, orthopedics, rehabilitation

## Abstract

Infrared radiation (IR) is a promising complementary treatment for musculoskeletal conditions and chronic pain. By means of a systematic review, we evaluated the contribution of IR to the management of these ailments. PubMed-MEDLINE, Scopus, and Cochrane Library–Cochrane Central Register of Controlled Trials were systematically searched until 20 December 2021. The literature search yielded 233 relevant records. Following the screening of titles and abstracts, 42 full-texts were evaluated. As per inclusion/exclusion criteria, 13 publications were entered into the qualitative assessment. These studies described the effects of IR in humans: three studies focused on osteoarthritis, four studies on fibromyalgia, and six encompassed a wider range of diseases (ankylosing spondylitis, recovery from sports injuries, myofascial pain syndrome). Based on the findings of our systematic review, we noted a decrease in pain levels, as evaluated by the visual analog scale (VAS), in patients suffering from musculoskeletal disorders treated with IR. In addition, IR use led to a decrease in Fibromyalgia Impact Questionnaire (FiQ) scores in subjects diagnosed with fibromyalgia. Nevertheless, IR has failed to facilitate muscle recovery following athletic injuries.

## 1. Introduction

The effort to treat musculoskeletal conditions with physical means is as old as humanity [1]. During the last decades, a growing body of research has focused on the use of infrared radiation (IR). IR, also known as infrared light, consists of electromagnetic radiation with wavelengths but shorter than microwave radiation and longer than visible light (750 nm to 1 mm). IR derives from thermal energy; hence, material bodies emitting heat can produce it. Higher temperatures lead to the production of higher amounts of IR of higher frequency and shorter wavelength [2]. The International Commission on Illumination (CIE) has divided IR into three bands according to its physical properties; near-infrared (IR-A, 700–1400 nm), mid-infrared (IR-B, 1400–3000 nm), and far-infrared (IR-C, 3000 nm–0.1 mm) [3,4].

### 1.1. Production and Provision of IR in Clinical Settings

IR for clinical use can be produced by luminous and non-luminous generators [3]. In the first case, a pre-heated wire in a non-light emitting lamp is used as a generator. In the second case, a lamp is used to produce a combination of infrared, visible, and ultraviolet light. Visible and ultraviolet light are filtered on the surface of the lamp [3]. The penetration of IR to the human body varies depending on the wavelength of infrared light and to the structure, vasculature, and pigmentation of the skin. The shorter the wavelength, the greater the penetration depth; in particular, IR-A can penetrate up to 5 mm. IR-C and IR-B have smaller penetration depths [5]. Although IR can directly reach only the epidermis, mechanical conduction of heat enables it to affect deeper layers of the skin and the fascia, with some textbooks reporting a penetration depth of up to 4 cm [3].

IR therapies can be performed with luminous or non–luminous lamps, clothing with IR–emitting ceramics, and IR saunas. Depending on their intensity, lamps need to be placed at a distance between 45 and 75 cm from the body. Exposure should be limited to 10–15 min, and eye coverage is recommended [3]. Tourmaline and nephrite-based ceramics have been used by patients with chronic pain or elite athletes [5], while far-infrared saunas entail 15 min long rest in a heated chamber (60 °C) followed by 40 min long recumbency and wrapping in thermal blankets [4].

### 1.2. Effects of Infrared Light on Health

From a mechanistic point of view, IR can affect living tissues from cellular to organ system level. At a molecular level, IR affects the rotation of molecules contained in body fluids and tissues. The potential clinical and physiological effect depends on the composition of the tissue and the proportion of biomolecules contained in body fluids [6,7]. At the cellular level, the interaction between IR and living tissue boils down to heat–induced alterations of cell membrane potentials by means of a rise in intracellular Ca^2+^ levels. This occurs due to increased membrane permeability for Ca^2+^ and increased Ca^2+^ release from the endoplasmic reticulum as a response to reactive oxygen species (ROS) generated because of the exposure of cells to radiation [8,9,10]. At the tissue level, a rise in the levels of Nitric Oxide (NO), an endogenous antioxidant substance countering the production of ROS, is associated with oxidative stress reduction and vasodilation. Simultaneously, it stimulates growth factor production and extracellular matrix deposition promoting tissue repair [11]. In this sense, IR-associated improvement in blood circulation can promote injury and pressure sores healing, decrease muscle spasms and improve the sensory nerve conduction velocity, and potentially increase endorphins modulating pain—the latter might be regulated by other factors as well [6]. At large, exposure to IR has been considered as an analog of exercise conveying similar benefits to either athletes or patients receiving IR sessions. Certainly, several aspects of the interaction of IR with the neuromusculoskeletal tissue are yet to be clarified.

**Figure 1:**
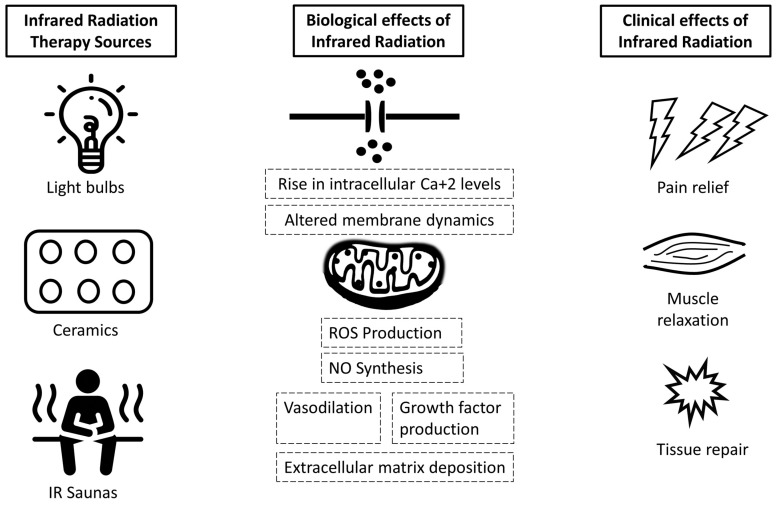
Schematic presentation of IR therapy means of treatment and hypothesized biological and clinical effects with a focus on the musculoskeletal system. Briefly, exposure to infrared radiation leads to an intracellular increase in reactive oxygen species (ROS) and a subsequent rise in nitric oxide (NO) synthesis and calcium intracellular levels (Ca^2+^). Eventually, this decreases oxidative stress, induces vasodilation and stimulates growth factor production and extracellular matrix deposition leading to tissue repair. Abbreviations: reactive oxygen species: ROS, nitric oxide: NO, calcium: Ca^2+^.

A summary of the hypothesized and investigated biological and clinical effects of IR therapy with a focus on the musculoskeletal system is presented in Figure 1.

IR consists of a promising complementary treatment for a wide range of musculoskeletal conditions, including knee osteoarthritis, fibromyalgia, chronic low back pain, chronic myofascial syndrome, sacroiliitis, and Gulf War Illness [8,9,10,11,12,13,14,15,16,17,18,19,20]. Infrared therapy can be delivered with light-emitting devices (photobiomodulation), wires, ceramic materials, and IR saunas. In a number of studies, infrared therapy has also been associated with clinical and perceptual improvement in chronic-pain-related psychosomatic symptoms such as fatigue and insomnia [12,13,14,15,16,17,18,19,20,21,22,23,24]. By means of a systematic review, we aimed to evaluate the use of infrared radiation in the management of musculoskeletal conditions and chronic pain.

## 2. Materials and Methods

### 2.1. Literature Search

**Figure 2:**
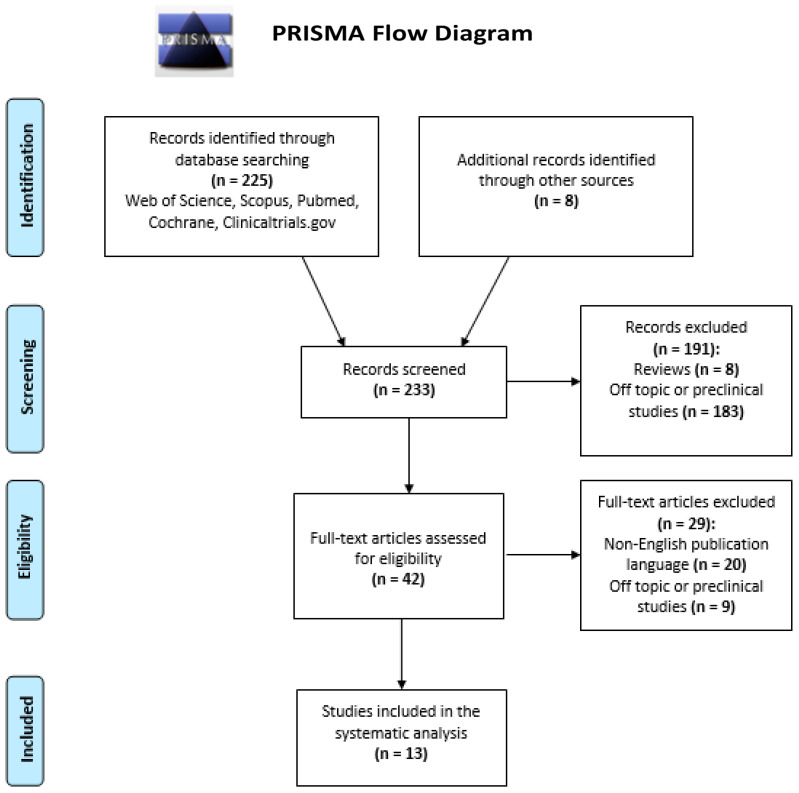
PRISMA Literature search flow diagram. From: Moher D, Liberati A, Tetzlaff J, Altman DG, The PRISMA Group (2009) [25,26].

To identify relevant peer-reviewed publications and gray literature, we searched PubMed-Medline, Scopus, and Cochrane Library–Cochrane Central Register of Controlled Trials (CENTRAL) till 20 December 2021. The reference lists of the selected sources and relevant systematic reviews were also hand-searched to identify potentially relevant resources. The search terms (infrared photobiomodulation, infrared therapy, infrared rays[MeSH] musculoskeletal diseases[MeSH], musculoskeletal pain[MeSH]) were used in combination with Boolean operators (AND, OR), when appropriate. Studies were included if they fulfilled all the following eligibility criteria: (1) ongoing or published clinical studies reporting on infrared therapeutic interventions for musculoskeletal conditions and musculoskeletal pain (2) epidemiological analyses and reports. A study was excluded if it met at least one of the following criteria: (1) non-English publication language, (2) study types: editorials, opinion articles, perspectives, and letters to the editor. No sample size restriction was applied when screening for eligible studies. Disputes in the selection of relevant studies were discussed between the two primary authors and a senior author until a consensus was reached. The literature was searched and reported according to the Preferred Reporting Items for Systematic Reviews and Meta-Analysis (PRISMA) extension for Scoping Reviews (PRISMASc) [25,26]. The search strategy is illustrated in Figure 2.

### 2.2. Data Synthesis and Analysis

After gathering information on eligible study design, sample size, population characteristics, duration of follow-up, and outcomes of interest, we aimed to quantify the post-intervention effect of IR-based treatment on self-reported measures of quality of life, as assessed in each study included. Those data should be reported for both control and intervention groups of participants as means or medians with corresponding standard deviations or quartiles.

## 3. Results

The literature search yielded 233 relevant records. Following titles and abstracts screening, the full-texts of 42 publications were evaluated. As per inclusion and exclusion criteria, 13 publications were included in the study and screened for comparable data eligible for further qualitative analysis. A number of primary studies describe its effects on humans. In particular, three studies focused on osteoarthritis, four studies on fibromyalgia, and six encompassed a wider range of diseases (ankylosing spondylitis, recovery from sports injuries, myofascial pain syndrome) [12,13,14,15,16,17,18,19,20,21,22,23,24]. A detailed account of the results of these studies is presented in Table 1.

### 3.1. Qualitative Outcomes

#### 3.1.1. Osteoarthritis

Three studies, two randomized control trials, and one observational study reported the use of IR in the management of osteoarthritis. The studies were published between 2012 and 2019 and included a total of 276 patients. These studies examined the efficacy of infrared energy-treatment on patients with osteoarthritis by means of questionnaires and clinical assessment methods. Hsieh et al. employed therapy with short-term monochromatic infrared energy and evaluated its impact on the Knee injury and Osteoarthritis Outcome Score, the Lysholm Knee Scale, Hospital Anxiety and Depression Scale, the Multidimensional Fatigue Inventory, and the Chronic Pain Grade questionnaire [13]. In the study by Bagnato et al., the primary endpoint was to assess pain improvement from baseline to 1-month post-treatment using the visual analog score (VAS) [14], whereas Lin et al. used WOMAC index scores and SF-36 scores in order to assess the severity of symptoms [20]. Hsieh and Lin concluded that there was no significant effect on patients [13,24]. However, Bagnato showed that far-infrared emitting plasters could be considered as an effective short–term non-pharmacological choice for the therapeutic management of knee osteoarthritis, as there was a 12.5% improvement (after 1 week) and a 25% (after 4 weeks). Bagnato suggests that IR could be beneficial as a complementary therapy for patients with osteoarthritis. However, further randomized controlled trials are needed in order to further elucidate the biological effect of FIR in osteoarthritis symptoms and clinical courses. The use of larger sample sizes, longer treatment duration, and higher photon energy levels is also recommended [14].

#### 3.1.2. Fibromyalgia

Three Randomized controlled trials and one observational study focused on fibromyalgia (FM). These studies were published between 2007 and 2019 and included 219 patients. All studies reported significant pain and FM symptoms reduction and quality of life improvement. In particular, fabrics coated with bioceramics could decrease FM-associated pain and burden in female patients by 21% in the VAS and FIQ assessment scales [17]. WAON therapy (soothing warmth therapy) was associated with an even greater improvement of FM symptoms assessed by both scales (52.7% in VAS, 34.6% in FIQ) in both male and female patients [16]. Similar results were reported with the use of AE and FIR [16] and mild water-filtered near-infrared whole-body hyperthermia (NI-WBH) [15] in both male and female patients. The latter appeared as a beneficial addition to standard multimodal rehabilitation (MR) sessions [15]. FIR treatment sessions were provided for 2–6 weeks [16,18], and patients were monitored for a time period ranging between 2 weeks and 6 months [15]. The reported relief was maintained during the monitoring period [18] or was significant within the first 6 weeks post-treatment [15].

#### 3.1.3. Other Conditions

Several studies have evaluated the effect of IR on a diverse set of musculoskeletal conditions [12,20,21,22,23] or occupational statuses [19]. The investigated variants of IR treatment included photobiomodulation (wIRA, MIPE, transcranial NIR, photobiomodulation), IR patches [21], and IR saunas [20] and appeared to decrease pain, fatigue, and/or insomnia in patients with sacroiliitis in the context of ankylosing spondylitis [12], myofascial pain syndrome [21], chronic fatigue syndrome [20] and Gulf War Illness [23] and chronic low back pain [22]. With regard to the latter, MIPE appeared to be approximately 25% more effective than laser phototherapy. Contrary to these, Guimarães et al. (2021) reported no significant difference between IR therapy and placebo within 1 year of monitoring in patients with low back pain [27]. Four studies were designed as randomized controlled trials and included relatively large patient groups ranging between 70 and 148 patients [12,21,22,27]. The remaining studies included 11 and 2 patients. Monitoring time ranged from days [12,20,21] and 6 and 12 weeks to 12 months [22,23].

Two additional studies focused on athletic injuries. FIR emitting ceramic clothing in athletes showed no short-term (72 h) benefit with regard to exercise-induced muscle damage. This evaluation included biochemical (creatinine kinase, lactate dehydrogenase), neuromuscular and perceptual (muscle soreness, maximal voluntary contraction, etc.) variables [19]. On the contrary, 830 nm phototherapy was associated with a significant decrease in pain, and 50% accelerated return-to-play (RTP) time in university athletes treated for 53 injury types with knee sprains, hamstring strains, Achilles’ tendonitis, intercostal strains, and shoulder sprains being the most prevalent. Overall, it appears that IR was beneficial with regard to relieving patients with chronic pain, fatigue, and related psychosomatic symptoms but had ambivalent efficacy with regard to sports injuries [19].

In terms of quantitative outcomes, as described in Table 1, the use of IR led to significant improvement of patient symptoms since the measures of quality of life assessed seem to be substantially decreased. There was a decrease in pain levels, as measured using the visual analog scale (VAS) of pain, in patients suffering from musculoskeletal disorders and being treated with IR-based therapy. Moreover, IR therapy decreased Fibromyalgia Impact Questionnaire (FiQ) scores in patients suffering from fibromyalgia. Future research should focus on the quantitative assessment of these findings by means of a meta-analysis.

## 4. Discussion

This systematic review of existing studies showed that IR has significant potential in reducing musculoskeletal pain. The majority of the reported studies suggest that IR could be beneficial as a complementary therapy for patients with FM, osteoarthritis, and other musculoskeletal conditions and athletic injuries associated with chronic pain and physical and psychological burden. IR efficacy in low back pain is ambivalent, with IR appearing more effective than low-level laser therapy in the treatment of chronic low back pain [18], but not significantly better than moxibustion therapy [24] and placebo visual-wavelength phototherapy [27]. The latter was observed in a one-year-long RCT involving a double-sized patient population, and, therefore, it creates considerable concerns about the efficacy of IR in low back pain [27].

The role of IR in facilitating recovery following sports injuries was also ambivalent. The clinical, neuromuscular, and biochemical findings of Nunes et al. [19] were in line with recent analyses focusing on phototherapy in sports injuries [28]. This was in contrast to the findings of Foley et al. that indicated IR phototherapy as an effective means of pain relief and RTP time acceleration. Although a definitive comparison is not feasible, it seems that regular phototherapy sessions are preferable to ceramic clothing [29].

The majority of the included studies focused on phototherapy and IR emitting ceramic materials, while fewer studies reported on IR saunas. Given the heterogeneity in methods and health conditions, it is not possible to reach a definite conclusion regarding the preferred IR treatment means. The heterogeneity of population sizes and characteristics does not lead to decisive evidence about age, gender, or patient groups that can benefit more from IF therapy.

Previous research has expressed safety concerns, implying that IR may be associated with thermal burns, skin irritation, eye damage, dehydration, hypotension, and defective arterial blood flow. On these grounds, IR has been contraindicated in patients with impaired cutaneous thermal sensations, superficial circulation defects, dermatitis or eczema, cancer, ionizing–radiation-associated skin damage, fever, and active infections [30,31,32]. In the present review, both the studies that showed the effectiveness or the supremacy of IR over other treatments and those that considered IR inferior to other therapeutic modalities did not express concerns regarding its safety. Previous research has identified pain, burn injury, and infection as key safety concerns [33]. Nevertheless, such adverse effects were not documented in the studies that we reported. Therefore, it appears that IR is safe in the above-mentioned conditions and delivery methods. These results encourage the clinical use of IR and urge for larger studies focusing on patients rather than healthy individuals in order to outline the indications and the expected benefits from the use of IR.

From a methodological point of view, the majority of the studies employed perceptual and clinical indexes in order to measure the effectiveness of IR, with only a few studies involving biochemical and/or neuromuscular parameters [19]. In most cases, IR was evaluated as a complementary therapy, and the conventional treatment of the reported patients was not discontinued. Although this can be considered as a source of controversy, pre- and post-treatment measurements served as a comparison that elucidated the effect of IR therapy. On top of this, discontinuing the mainstay treatment of patients with chronic conditions might not have been possible on ethical grounds.

### 4.1. Limitations

The included studies had several limitations. The limited sample size, in combination with the lack of long-term monitoring, can cast doubt on the real-world significance of the reported outcomes. As a matter of fact, although Matsushita et al. reported remarkable results—namely, improvement by up to 50% in pain assessment scores—their study included only 13 patients [18]. The use of common reporting systems and scales was beneficial with regard to the comparison of the studies and the different FIR treatment methods; however, long-term follow-up is necessary. Most significantly, we could not quantify the effect of IR therapy on every study included since we could not extract the required numerical data for both experimental and control study groups.

### 4.2. Future Research

Future research should include a wider range of assessment tools evaluating patients’ functionality and quality of life on top of a decrease in pain and reported symptoms. Including data from a patient’s follow-up imaging and laboratory examinations can help understand whether FIR-based treatments have the potential to delay the progression of degenerative conditions such as osteoarthritis. Blinding these studies remains challenging, given that patients receive or are already aware of information that allows them to understand whether they belong to the study or the control group. Research in the field of musculoskeletal pain remains important as it can manifest as early as adolescence. For example, schoolchildren often experience musculoskeletal pain due to the weight of their schoolbags [34,35].

## 5. Conclusions

Overall, IR appears as a safe and effective complementary therapy for a number of musculoskeletal conditions, including knee OA, FM, and chronic myofascial pain. Its efficacy remains debatable when it comes to low back pain and muscle damage in sports injuries. Given the diversity of therapeutic means and applications, further research is necessary in order to establish the optimal indications and treatment plan for the clinical use of IR in musculoskeletal medicine.

## Figures and Tables

**Table 1:** Characteristics and summarization of the included studies.

Disease	Study Type	Country	Treatment	Population (Intervention/Control)	Outcome Measurements	Key Outcomes	Reference
AS	RCT	China	wIRA	120 (60/60)	BASDAI, pain VAS, morning stiffness VAS, RI	↓ BASDAI ↓ pain VAS (↓ 17.5%) ↓ morning stiffness VAS ↑ RI	Xu/2019 [12]
Knee OA	RCT	Taiwan	MIRE Λ = 890 nm	73 (38/38)	KOOS, LKS, HADS, OAQoL	OAQoL ↓ 14.3%	Hsieh/2012 [13]
	RCT	Italy	FIR emitting plaster	60 (30/30)	pain VAS	↓ pain VAS 1 week: ↓12.5% 4 weeks: ↓25%	Bagnato/2012 [14]
	RCT pooled data	China	10.6 μm infrared laser vs. traditional moxibustion	143 (55/88)	WOMAC SF-36	pain VAS: ↓ 60.9% no significant differences	Lin/2020 [24]
FM	RCT	Germany	mild water-filtered NI-WBH	139 (69/70)	MPQ, FIQ	VAS: ↓ 37.5% FIQ: ↓ 40.4%	Brockow/2007 [15]
	RCT	Brazil	AE, FIR	28 (14/14)	pain VAS SF-MPQ FIQ	VAS: ↓ 41.4% FIQ: ↓ 14.7%	Salm/2019 [16]
	RCT	Spain	fabric coated with bio-ceramics	39 (20/19)	pain VAS FIQ SF-12	VAS: ↓21.76% FIQ: ↓16.46%	Campos/2017 [17]
	OS	Japan	Waon therapy	13	pain VAS FIQ	VAS: ↓52.7% FIQ: ↓34.6%	Matsushita/2008 [18]
Injuries (athletes)	RCT	Brazil	cFIR	20 (10/10)	performance biochemical markers delayed-onset muscle soreness training strain	↑ recovery	Nunes/2020 [19]
	OS	Japan	Waon therapy	11	PSS	↓ perceived fatigue ↑ performance status PSS: ↓19.23%	Soejima/2015 [20]
MPS	RCT	Taiwan	FIR patches	125 (61/57)	pain VAS pressure pain threshold maximal pain tolerance	pain VAS: ↓8.2% ↓ pressure pain threshold ↓ maximal pain tolerance	Lai/2017 [21]
CLBP	RCT	Egypt	MIPE vs. LLLT	70 (35/35)	FRI pain VAS modified Schober test	MIPE: 36% improvement LLLT: 27.87% improvement	Ammar/2015 [22]
Gulf War Illness	Case series	USA	NTIP	2	KGWMHHQ ISI BPI	pain, sleep, insomnia improvements	Chao/2019 [23]

*Legend:* AE, aquatic exercise. AS, ankylosing spondylitis. BASDAI, Bath ankylosing spondylitis disease activity index. BPI, and Brief Pain Inventory. cFIR, far-infrared emitting ceramic materials. CLBP, chronic low back pain. FIQ, FM Impact Questionnaire. FIR, Far infrared. FM, fibromyalgia. FRI, Functional rating index. HADS, Hospital Anxiety and Depression Scale. ISI, Insomnia Severity Index. KGWMHHQ, Kansas Gulf War Military History and Health Questionnaire. KOOS, knee injury and Osteoarthritis Outcome Score. LKS, Lysholm Knee Scale. LLLT, Low Level Laser Therapy. MIPE, Monochromatic Infrared Photo Energy. MIRE, monochromatic infrared energy-treatment. MPQ, McGill Pain Questionnaire. MPS, myofascial pain syndrome. NI-WBH, near infrared whole-body hyperthermia. NTIP, Near-infrared transcranial and intranasal photobiomodulation. nm, nanometers. OA, osteoarthritis. OAQoL, OA Quality of Life Questionnaire. OS, observational study. PSS, pain self-rate scale. RCT, randomized placebo-controlled trial. RI, resistance index. SF-12, Short Form-12 health survey. SF-36, Medical Study 36-item Short-Form Health Survey. SF-MPQ, short form of McGill Pain Questionnaire. VAS, pain-visual analogue scale. wIRA, water-filtered infrared-A-radiation. WOMAC, Western Ontario and McMaster Universities Osteoarthritis Index. ↑, increase. ↓, decrease. Λ, wavelength.

## Data Availability

Not applicable.
